# Supplementary material for: New insights into anatomical connectivity along the anterior–posterior axis of the human hippocampus using in vivo quantitative fibre tracking
Source: eLife. 2022 Nov 8;11:e76143. doi: 10.7554/eLife.76143 (PMC9643002; doi:10.7554/eLife.76143)
Supplement: Supplementary file 1. — Connectivity values between the whole hippocampus and all cortical areas in the Human Connectome Project Multi-Modal Parcellation (HCPMMP) scheme are presented. Column 1 displays each broad brain region ordered by their strength of connectivity with the whole hippocampus. Column 2 displays the percent of all cortical connections accounted for by each region. Values in brackets indicate the percent of cortical connections accounted for by each region when excluding medial temporal lobe (MTL) areas. Column 3 presents the cortical areas located within each broad brain region ordered by their strength of connectivity with the whole hippocampus (abbreviations for all cortical areas are defined in Supplementary file 3). Column 4 displays the mean SIFT2 weighted value (connectivity strength) associated with each cortical area. Column 5 displays the standard error of the mean. Column 6 displays the percent of all cortical connections accounted for by each cortical area. Values in brackets indicate the percent of cortical connections accounted for by each cortical area when excluding MTL areas. Column 7 displays the rank order for each cortical area by strength of connectivity. Values in brackets indicate the rank order when excluding MTL areas. [file elife-76143-supp1.docx]

**Supplementary File 1. Connectivity values between the cortical mantle and the whole hippocampus.** Connectivity values between the whole hippocampus and all cortical areas in the Human Connectome Project Multi-Modal Parcellation (HCPMMP) scheme are presented. Column 1 displays each broad brain region ordered by their strength of connectivity with the whole hippocampus. Column 2 displays the percent of all cortical connections accounted for by each region. Values in brackets indicate the percent of cortical connections accounted for by each region when excluding medial temporal lobe (MTL) areas. Column 3 presents the cortical areas located within each broad brain region ordered by their strength of connectivity with the whole hippocampus (abbreviations for all cortical areas are defined in Table S3). Column 4 displays the mean SIFT2 weighted value (connectivity strength) associated with each cortical area. Column 5 displays the standard error of the mean. Column 6 displays the percent of all cortical connections accounted for by each cortical area. Values in brackets indicate the percent of cortical connections accounted for by each cortical area when excluding MTL areas. Column 7 displays the rank order for each cortical area by strength of connectivity. Values in brackets indicate the rank order when excluding MTL areas.

| **Brain region** | **Percent of all cortical connections accounted for by region.**  **(Percent of cortical connections excluding medial temporal areas)** | **Cortical areas within each brain region** | **Mean SIFT2 weighted value (connectivity strength; n=10)** | **SE of mean** | **Percent of all cortical connections accounted for by area.**  **(Percent of cortical connections excluding MTL areas)** | **Rank order for each cortical area by strength of connectivity**  **(Rank order for each cortical area excluding MTL areas)** |
| --- | --- | --- | --- | --- | --- | --- |
| Medial Temporal Cortex | 52.0  (N/A) | EC | 35585 | 3509 | 23.64 (N/A) | 1 (N/A) |
|  |  | PeEc | 21683 | 2377 | 14.40 (N/A) | 2 (N/A) |
|  |  | PHA2 | 8603 | 854 | 5.72 (N/A) | 3 (N/A) |
|  |  | PHA1 | 7902 | 887 | 5.25 (N/A) | 4 (N/A) |
|  |  | PHA3 | 4447 | 359 | 2.95 (N/A) | 8 (N/A) |
| Lateral Temporal Cortex (including Temporal Pole) | 13.3  (27.8) | TF | 7673 | 886 | 5.10 (10.61) | 5 (1) |
|  |  | TGd | 3465 | 288 | 2.30 (4.79) | 11 (6) |
|  |  | TGv | 3337 | 313 | 2.22 (4.61) | 12 (7) |
|  |  | TE2a | 2214 | 288 | 1.47 (3.06) | 14 (9) |
|  |  | TE2p | 1288 | 198 | 0.86 (1.78) | 23 (18) |
|  |  | TE1a | 694 | 96 | 0.46 (0.96) | 29 (24) |
|  |  | TE1p | 676 | 99 | 0.45 (0.93) | 30 (25) |
|  |  | TE1m | 461 | 77 | 0.31 (0.64) | 33 (28) |
|  |  | PHT | 265 | 34 | 0.18 (0.37) | 41 (36) |
| Medial Parietal Cortex (including Posterior Cingulate) | 11.3  (23.5) | ProS | 5483 | 784 | 3.64 (7.58) | 6 (2) |
|  |  | POS1 | 3712 | 424 | 2.47 (5.13) | 10 (5) |
|  |  | RSC | 2063 | 121 | 1.37 (2.85) | 16 (11) |
|  |  | DVT | 1939 | 335 | 1.29 (2.68) | 18 (13) |
|  |  | POS2 | 1802 | 248 | 1.20 (2.49) | 19 (14) |
|  |  | 7m | 696 | 96 | 0.46 (0.96) | 28 (23) |
|  |  | v23ab | 565 | 85 | 0.38 (0.78) | 31 (26) |
|  |  | d23ab | 178 | 12 | 0.12 (0.25) | 55 (50) |
|  |  | 31pd | 156 | 22 | 0.10 (0.22) | 62 (57) |
|  |  | PCV | 130 | 24 | 0.09 (0.18) | 66 (61) |
|  |  | 31pv | 87 | 8 | 0.06 (0.12) | 74 (69) |
|  |  | 23d | 47 | 6 | 0.03 (0.06) | 89 (84) |
|  |  | 23c | 42 | 5 | 0.03 (0.06) | 93 (88) |
|  |  | 31a | 41 | 5 | 0.03 (0.06) | 94 (89) |
| Early Visual Cortex (Occipital) | 9.2  (19.2) | V1 | 5385 | 579 | 3.58 (7.45) | 7 (3) |
|  |  | V2 | 3840 | 462 | 2.55 (5.31) | 9 (4) |
|  |  | V3 | 3079 | 450 | 2.05 (4.26) | 13 (8) |
|  |  | V4 | 1601 | 127 | 1.06 (2.21) | 22 (17) |
| Ventral Stream Visual Cortex | 6.1  (12.7) | VMV2 | 2105 | 247 | 1.40 (2.91) | 15 (10) |
|  |  | VVC | 1956 | 220 | 1.30 (2.71) | 17 (12) |
|  |  | VMV1 | 1788 | 248 | 1.19 (2.47) | 20 (15) |
|  |  | FFC | 1670 | 172 | 1.11 (2.31) | 21 (16) |
|  |  | VMV3 | 819 | 34 | 0.54 (1.13) | 26 (21) |
|  |  | V8 | 516 | 53 | 0.34 (0.71) | 32 (27) |
|  |  | PIT | 326 | 51 | 0.22 (0.45) | 40 (35) |
| Dorsal Stream Visual Cortex | 2.2  (4.5) | V6 | 1050 | 195 | 0.70 (1.45) | 24 (19) |
|  |  | V3A | 1029 | 197 | 0.68 (1.42) | 25 (20) |
|  |  | IPS1 | 358 | 37 | 0.24 (0.50) | 37 (32) |
|  |  | V7 | 334 | 40 | 0.22 (0.46) | 38 (33) |
|  |  | V6A | 331 | 52 | 0.22 (0.46) | 39 (34) |
|  |  | V3B | 176 | 23 | 0.12 (0.24) | 58 (53) |
| MT+ Complex and Neighbouring Visual Areas | 1.5  (3.2) | PH | 779 | 86 | 0.52 (1.08) | 27 (22) |
|  |  | V3CD | 234 | 31 | 0.16 (0.32) | 45 (40) |
|  |  | FST | 231 | 25 | 0.15 (0.32) | 46 (41) |
|  |  | LO2 | 201 | 33 | 0.13 (0.28) | 49 (44) |
|  |  | LO3 | 183 | 22 | 0.12 (0.25) | 51 (46) |
|  |  | V4t | 182 | 31 | 0.12 (0.25) | 52 (47) |
|  |  | MST | 177 | 21 | 0.12 (0.24) | 56 (51) |
|  |  | MT | 177 | 22 | 0.12 (0.24) | 57 (52) |
|  |  | LO1 | 132 | 24 | 0.09 (0.18) | 65 (60) |
| Inferior Parietal Cortex | 1.0  (2.0) | PGp | 414 | 33 | 0.28 (0.57) | 34 (29) |
|  |  | PGs | 249 | 36 | 0.17 (0.34) | 43 (38) |
|  |  | PGi | 181 | 21 | 0.12 (0.25) | 53 (48) |
|  |  | PFm | 179 | 27 | 0.12 (0.25) | 54 (49) |
|  |  | IP0 | 173 | 14 | 0.11 (0.24) | 60 (55) |
|  |  | IP1 | 118 | 11 | 0.08 (0.16) | 68 (63) |
|  |  | PF | 59 | 10 | 0.04 (0.08) | 81 (76) |
|  |  | IP2 | 33 | 6 | 0.02 (0.05) | 105 (100) |
|  |  | PFt | 13 | 2 | 0.01 (0.02) | 126 (121) |
|  |  | PFop | 10 | 1 | 0.01 (0.01) | 132 (127) |
| Auditory Association Cortex | 0.9  (1.8) | STSva | 403 | 40 | 0.27 (0.56) | 36 (31) |
|  |  | STSvp | 238 | 30 | 0.16 (0.33) | 44 (39) |
|  |  | STSda | 195 | 21 | 0.13 (0.27) | 50 (45) |
|  |  | STSdp | 176 | 34 | 0.12 (0.24) | 59 (54) |
|  |  | STGa | 96 | 13 | 0.06 (0.13) | 71 (66) |
|  |  | A5 | 91 | 14 | 0.06 (0.13) | 73 (68) |
|  |  | A4 | 78 | 13 | 0.05 (0.11) | 76 (71) |
|  |  | TA2 | 33 | 6 | 0.02 (0.05) | 106 (101) |
| Insular and Frontal Opercular Cortex | 0.7  (1.5) | PoI1 | 408 | 86 | 0.27 (0.56) | 35 (30) |
|  |  | Pir | 259 | 37 | 0.17 (0.36) | 42 (37) |
|  |  | PI | 202 | 28 | 0.13 (0.28) | 48 (43) |
|  |  | 52 | 99 | 12 | 0.07 (0.14) | 70 (65) |
|  |  | AAIC | 36 | 6 | 0.02 (0.05) | 100 (95) |
|  |  | PoI2 | 36 | 8 | 0.02 (0.05) | 101 (96) |
|  |  | Ig | 6 | 1 | < 0.01 (0.01) | 145 (140) |
|  |  | MI | 6 | 2 | < 0.01 (0.01) | 146 (141) |
|  |  | FOP4 | 4 | 1 | < 0.01 (0.01) | 156 (151) |
|  |  | AVI | 3 | 1 | < 0.01 (< 0.01) | 163 (158) |
|  |  | FOP5 | 2 | 1 | < 0.01 (< 0.01) | 170 (165) |
|  |  | FOP3 | 2 | 0 | < 0.01 (< 0.01) | 171 (166) |
|  |  | FOP2 | 2 | 0 | < 0.01 (< 0.01) | 172 (167) |
| Superior Parietal Cortex | 0.7  (1.4) | 7Pm | 216 | 41 | 0.14 (0.30) | 47 (42) |
|  |  | 7PL | 160 | 27 | 0.11 (0.22) | 61 (56) |
|  |  | MIP | 149 | 20 | 0.10 (0.21) | 63 (58) |
|  |  | 7Am | 139 | 23 | 0.09 (0.19) | 64 (59) |
|  |  | VIP | 81 | 13 | 0.05 (0.11) | 75 (70) |
|  |  | 7AL | 78 | 15 | 0.05 (0.11) | 77 (72) |
|  |  | LIPv | 73 | 14 | 0.05 (0.10) | 79 (74) |
|  |  | 7PC | 48 | 9 | 0.03 (0.07) | 87 (82) |
|  |  | LIPd | 38 | 7 | 0.03 (0.05) | 99 (94) |
|  |  | AIP | 20 | 3 | 0.01 (0.03) | 116 (111) |
| Temporo-Parieto-Occipital Junction | 0.3  (0.6) | TPOJ3 | 130 | 12 | 0.09 (0.18) | 67 (62) |
|  |  | TPOJ2 | 104 | 11 | 0.07 (0.14) | 69 (64) |
|  |  | TPOJ1 | 96 | 13 | 0.06 (0.13) | 72 (67) |
|  |  | PSL | 51 | 5 | 0.03 (0.07) | 83 (78) |
|  |  | STV | 48 | 6 | 0.03 (0.07) | 88 (83) |
| Anterior Cingulate and Medial Prefrontal Cortex | 0.3  (0.6) | 33pr | 75 | 10 | 0.05 (0.10) | 78 (73) |
|  |  | a24pr | 49 | 6 | 0.03 (0.07) | 84 (79) |
|  |  | p32pr | 49 | 8 | 0.03 (0.07) | 85 (80) |
|  |  | a32pr | 46 | 8 | 0.03 (0.06) | 90 (85) |
|  |  | 8BM | 41 | 6 | 0.03 (0.06) | 95 (90) |
|  |  | p24pr | 41 | 4 | 0.03 (0.06) | 96 (91) |
|  |  | p24 | 35 | 6 | 0.02 (0.05) | 102 (97) |
|  |  | 9m | 31 | 6 | 0.02 (0.04) | 109 (104) |
|  |  | d32 | 28 | 5 | 0.02 (0.04) | 111 (106) |
|  |  | a24 | 14 | 3 | < 0.01 (0.02) | 125 (120) |
|  |  | p32 | 5 | 1 | < 0.01 (0.01) | 153 (148) |
|  |  | 10r | 3 | 0 | < 0.01 (< 0.01) | 164 (159) |
|  |  | 10v | 2 | 1 | < 0.01 (< 0.01) | 173 (168) |
|  |  | 25 | 2 | 1 | < 0.01 (< 0.01) | 174 (169) |
|  |  | s32 | 2 | 0 | < 0.01 (< 0.01) | 175 (170) |
| Somatosensory and Motor Cortex | 0.2  (0.3) | 4 | 62 | 9 | 0.04 (0.09) | 80 (75) |
|  |  | 2 | 55 | 8 | 0.04 (0.08) | 82 (77) |
|  |  | 1 | 39 | 7 | 0.03 (0.05) | 98 (93) |
|  |  | 3b | 33 | 5 | 0.02 (0.05) | 107 (102) |
|  |  | 3a | 21 | 4 | 0.01 (0.03) | 114 (109) |
|  |  | 5m | 11 | 2 | < 0.01 (0.02) | 129 (124) |
| Early Auditory Cortex | 0.1  (0.3) | PBelt | 49 | 8 | 0.03 (0.07) | 86 (81) |
|  |  | LBelt | 45 | 6 | 0.02 (0.06) | 91 (86) |
|  |  | MBelt | 43 | 7 | 0.02 (0.06) | 92 (87) |
|  |  | RI | 35 | 3 | 0.02 (0.05) | 103 (98) |
|  |  | A1 | 30 | 4 | 0.02 (0.04) | 110 (105) |
| Paracentral Lobular and Mid Cingulate Cortex | 0.1  (0.3) | 5mv | 40 | 6 | 0.03 (0.06) | 97 (92) |
|  |  | 5L | 34 | 9 | 0.02 (0.05) | 104 (99) |
|  |  | SCEF | 32 | 5 | 0.02 (0.04) | 108 (103) |
|  |  | 6mp | 27 | 5 | 0.02 (0.04) | 112 (107) |
|  |  | 6ma | 26 | 4 | 0.02 (0.04) | 113 (108) |
|  |  | 24dv | 20 | 7 | 0.01 (0.03) | 117 (112) |
|  |  | 24dd | 17 | 3 | 0.01 (0.02) | 121 (116) |
| Dorsolateral Prefrontal Cortex | < 0.1  (0.2) | SFL | 21 | 3 | 0.01 (0.03) | 115 (110) |
|  |  | 8BL | 19 | 3 | 0.01 (0.03) | 118 (113) |
|  |  | 8Av | 17 | 3 | 0.01 (0.02) | 122 (117) |
|  |  | 8C | 10 | 2 | 0.01 (0.01) | 133 (128) |
|  |  | i6-8 | 10 | 2 | 0.01 (0.01) | 134 (129) |
|  |  | 9p | 9 | 2 | 0.01 (0.01) | 135 (130) |
|  |  | 9a | 8 | 1 | 0.01 (0.01) | 136 (131) |
|  |  | 9-46d | 7 | 1 | < 0.01 (0.01) | 140 (135) |
|  |  | 8Ad | 7 | 1 | < 0.01 (0.01) | 141 (136) |
|  |  | 46 | 7 | 1 | < 0.01 (0.01) | 142 (137) |
|  |  | s6-8 | 7 | 1 | < 0.01 (0.01) | 143 (138) |
|  |  | p9-46v | 6 | 1 | < 0.01 (0.01) | 147 (142) |
|  |  | a9-46v | 4 | 1 | < 0.01 (0.01) | 157 (152) |
| Premotor Cortex | < 0.1  (0.1) | 6r | 19 | 3 | 0.01 (0.03) | 119 (114) |
|  |  | 6a | 16 | 2 | 0.01 (0.02) | 123 (118) |
|  |  | 6d | 16 | 3 | 0.01 (0.02) | 124 (119) |
|  |  | 6v | 13 | 2 | 0.01 (0.02) | 127 (122) |
|  |  | FEF | 11 | 2 | 0.01 (0.02) | 130 (125) |
|  |  | 55b | 11 | 2 | 0.01 (0.02) | 131 (126) |
|  |  | PEF | 6 | 1 | < 0.01 (0.01) | 148 (143) |
| Orbital and Polar Frontal Cortex | < 0.1  (< 0.1) | 10d | 8 | 2 | 0.01 (0.01) | 137 (132) |
|  |  | a47r | 6 | 2 | < 0.01 (0.01) | 149 (144) |
|  |  | 47s | 6 | 1 | < 0.01 (0.01) | 150 (145) |
|  |  | 13l | 4 | 1 | < 0.01 (0.01) | 158 (153) |
|  |  | pOFC | 4 | 1 | < 0.01 (0.01) | 159 (154) |
|  |  | p10p | 4 | 1 | < 0.01 (0.01) | 160 (155) |
|  |  | OFC | 4 | 1 | < 0.01 (0.01) | 161 (156) |
|  |  | 10pp | 3 | 1 | < 0.01 (< 0.01) | 165 (160) |
|  |  | 11l | 3 | 1 | < 0.01 (< 0.01) | 166 (161) |
|  |  | a10p | 2 | 1 | < 0.01 (< 0.01) | 176 (171) |
|  |  | 47m | 2 | 0 | < 0.01 (< 0.01) | 177 (172) |
| Inferior Frontal Cortex | < 0.1  (< 0.1) | 44 | 12 | 2 | 0.01 (0.02) | 128 (123) |
|  |  | IFJa | 8 | 1 | 0.01 (0.01) | 138 (133) |
|  |  | IFSp | 7 | 1 | < 0.01 (0.01) | 144 (139) |
|  |  | IFSa | 5 | 1 | < 0.01 (0.01) | 154 (149) |
|  |  | 45 | 5 | 1 | < 0.01 (0.01) | 155 (150) |
|  |  | IFJp | 3 | 1 | < 0.01 (< 0.01) | 167 (162) |
|  |  | p47r | 3 | 1 | < 0.01 (< 0.01) | 168 (163) |
|  |  | 47l | 2 | 1 | < 0.01 (< 0.01) | 178 (173) |
| Posterior Opercular Cortex | < 0.1  (< 0.1) | PFcm | 18 | 3 | 0.01 (0.02) | 120 (115) |
|  |  | OP1 | 8 | 2 | 0.01 (0.01) | 139 (134) |
|  |  | OP4 | 6 | 1 | < 0.01 (0.01) | 151 (146) |
|  |  | 43 | 6 | 1 | < 0.01 (0.01) | 152 (147) |
|  |  | OP2-3 | 4 | 1 | < 0.01 (0.01) | 162 (157) |
|  |  | FOP1 | 3 | 1 | < 0.01 (< 0.01) | 169 (164) |
