# Supplementary material for: New insights into anatomical connectivity along the anterior–posterior axis of the human hippocampus using in vivo quantitative fibre tracking
Source: eLife. 2022 Nov 8;11:e76143. doi: 10.7554/eLife.76143 (PMC9643002; doi:10.7554/eLife.76143)
Supplement: Supplementary file 2. — Connectivity values between the head, body, and tail of the hippocampus and all cortical brain areas included in the Human Connectome Project Multi-Modal Parcellation (HCPMMP) scheme are presented. Column 1 displays each cortical area ordered by strength of connectivity with the whole hippocampus (abbreviations for all cortical areas are defined in Supplementary file 3). Column 2 presents the broader brain region within which each cortical area is located. Column 3 displays the mean SIFT2-weighted value (connectivity strength) associated with each cortical area and the head of the hippocampus. Column 4 displays the associated standard error of the mean. Column 5 displays the mean SIFT2-weighted value (connectivity strength) associated with each cortical area and the body of the hippocampus. Column 6 displays the associated standard error of the mean. Column 7 displays the mean SIFT2-weighted value (connectivity strength) associated with each cortical area and the tail of the hippocampus. Column 8 displays the associated standard error of the mean. [file elife-76143-supp2.docx]

**Supplementary File 2. Connectivity between the cortical mantle and the head, body and tail of the hippocampus.** Connectivity values between the head, body and tail of the hippocampus and all cortical brain areas included in the Human Connectome Project Multi-Modal Parcellation (HCPMMP) scheme are presented. Column 1 displays each cortical area ordered by strength of connectivity with the whole hippocampus (abbreviations for all cortical areas are defined in Table S3). Column 2 presents the broader brain region within which each cortical area is located. Column 3 displays the mean SIFT2 weighted value (connectivity strength) associated with each cortical area and the head of the hippocampus. Column 4 displays the associated standard error of the mean. Column 5 displays the mean SIFT2 weighted value (connectivity strength) associated with each cortical area and the body of the hippocampus. Column 6 displays the associated standard error of the mean. Column 7 displays the mean SIFT2 weighted value (connectivity strength) associated with each cortical area and the tail of the hippocampus. Column 8 displays the associated standard error of the mean.

| **Cortical area** | **Location of area** | **Head** | | **Body** | | **Tail** | |
| --- | --- | --- | --- | --- | --- | --- | --- |
|  |  | **Mean SIFT2 weighted value (n=10)** | **SE of Mean** | **Mean SIFT2 weighted value (n=10)** | **SE of Mean** | **Mean SIFT2 weighted value (n=10)** | **SE of Mean** |
| EC | Medial Temporal Cortex | 16206 | 1927 | 12679 | 1357 | 6700 | 843 |
| PeEc | Medial Temporal Cortex | 11144 | 1414 | 7838 | 998 | 2700 | 366 |
| PHA2 | Medial Temporal Cortex | 2644 | 322 | 4545 | 566 | 1414 | 116 |
| PHA1 | Medial Temporal Cortex | 2287 | 299 | 3716 | 450 | 1899 | 243 |
| TF | Lateral Temporal Cortex | 3287 | 500 | 3452 | 410 | 934 | 144 |
| ProS | Medial Parietal Cortex (including Posterior Cingulate) | 716 | 116 | 1625 | 194 | 3143 | 553 |
| V1 | Early Visual Cortex (Occipital) | 567 | 70 | 1389 | 112 | 3429 | 489 |
| PHA3 | Medial Temporal Cortex | 1420 | 134 | 2277 | 236 | 750 | 76 |
| V2 | Early Visual Cortex (Occipital) | 428 | 62 | 1054 | 82 | 2358 | 376 |
| POS1 | Medial Parietal Cortex (including Posterior Cingulate) | 620 | 89 | 1299 | 112 | 1793 | 297 |
| TGd | Lateral Temporal Cortex | 1576 | 182 | 1446 | 117 | 443 | 47 |
| TGv | Lateral Temporal Cortex | 1589 | 207 | 1358 | 144 | 389 | 41 |
| V3 | Early Visual Cortex (Occipital) | 327 | 37 | 810 | 83 | 1942 | 357 |
| TE2a | Lateral Temporal Cortex | 754 | 132 | 1038 | 107 | 422 | 65 |
| VMV2 | Ventral Stream Visual Cortex | 354 | 68 | 860 | 111 | 891 | 97 |
| RSC | Medial Parietal Cortex (including Posterior Cingulate) | 401 | 55 | 733 | 52 | 928 | 107 |
| VVC | Ventral Stream Visual Cortex | 460 | 76 | 969 | 129 | 528 | 48 |
| DVT | Medial Parietal Cortex (including Posterior Cingulate) | 247 | 36 | 515 | 65 | 1178 | 253 |
| POS2 | Medial Parietal Cortex (including Posterior Cingulate) | 303 | 32 | 590 | 47 | 909 | 195 |
| VMV1 | Ventral Stream Visual Cortex | 245 | 47 | 644 | 115 | 899 | 110 |
| FFC | Ventral Stream Visual Cortex | 355 | 49 | 798 | 109 | 517 | 55 |
| V4 | Early Visual Cortex (Occipital) | 183 | 20 | 472 | 40 | 946 | 116 |
| TE2p | Lateral Temporal Cortex | 348 | 69 | 658 | 101 | 282 | 45 |
| V6 | Dorsal Stream Visual Cortex | 113 | 16 | 259 | 37 | 678 | 149 |
| V3A | Dorsal Stream Visual Cortex | 103 | 11 | 252 | 35 | 674 | 155 |
| VMV3 | Ventral Stream Visual Cortex | 127 | 16 | 306 | 28 | 386 | 23 |
| PH | MT+ Complex and Neighbouring Visual Areas | 136 | 18 | 338 | 44 | 305 | 40 |
| 7m | Medial Parietal Cortex (including Posterior Cingulate) | 120 | 17 | 251 | 26 | 325 | 69 |
| TE1a | Lateral Temporal Cortex | 209 | 42 | 329 | 39 | 156 | 23 |
| TE1p | Lateral Temporal Cortex | 146 | 27 | 334 | 46 | 196 | 32 |
| v23ab | Medial Parietal Cortex (including Posterior Cingulate) | 91 | 14 | 207 | 23 | 267 | 60 |
| V8 | Ventral Stream Visual Cortex | 72 | 14 | 178 | 20 | 265 | 33 |
| TE1m | Lateral Temporal Cortex | 121 | 32 | 232 | 35 | 108 | 14 |
| PGp | Inferior Parietal Cortex | 58 | 7 | 147 | 12 | 209 | 25 |
| PoI1 | Insular and Frontal Opercular Cortex | 110 | 21 | 174 | 36 | 124 | 31 |
| STSva | Auditory Association Cortex | 108 | 17 | 198 | 17 | 98 | 9 |
| IPS1 | Dorsal Stream Visual Cortex | 38 | 6 | 100 | 6 | 220 | 27 |
| V7 | Dorsal Stream Visual Cortex | 38 | 6 | 88 | 6 | 207 | 32 |
| V6A | Dorsal Stream Visual Cortex | 45 | 8 | 92 | 14 | 194 | 33 |
| PIT | Ventral Stream Visual Cortex | 41 | 8 | 116 | 26 | 168 | 25 |
| PHT | Lateral Temporal Cortex | 52 | 10 | 127 | 15 | 86 | 13 |
| Pir | Insular and Frontal Opercular Cortex | 148 | 27 | 81 | 10 | 29 | 4 |
| PGs | Inferior Parietal Cortex | 37 | 8 | 100 | 13 | 112 | 17 |
| STSvp | Auditory Association Cortex | 46 | 8 | 122 | 15 | 70 | 9 |
| V3CD | MT+ Complex and Neighbouring Visual Areas | 20 | 3 | 65 | 7 | 149 | 26 |
| FST | MT+ Complex and Neighbouring Visual Areas | 34 | 5 | 92 | 13 | 105 | 14 |
| 7Pm | Superior Parietal Cortex | 43 | 9 | 77 | 16 | 96 | 19 |
| PI | Insular and Frontal Opercular Cortex | 55 | 12 | 89 | 9 | 58 | 9 |
| LO2 | MT+ Complex and Neighbouring Visual Areas | 23 | 3 | 65 | 11 | 113 | 24 |
| STSda | Auditory Association Cortex | 54 | 10 | 90 | 9 | 51 | 5 |
| LO3 | MT+ Complex and Neighbouring Visual Areas | 21 | 3 | 66 | 9 | 97 | 16 |
| V4t | MT+ Complex and Neighbouring Visual Areas | 24 | 5 | 62 | 13 | 96 | 18 |
| PGi | Inferior Parietal Cortex | 27 | 5 | 85 | 14 | 70 | 8 |
| PFm | Inferior Parietal Cortex | 27 | 6 | 80 | 11 | 73 | 12 |
| d23ab | Medial Parietal Cortex (including Posterior Cingulate) | 28 | 4 | 62 | 5 | 88 | 8 |
| MST | MT+ Complex and Neighbouring Visual Areas | 21 | 3 | 71 | 10 | 86 | 14 |
| MT | MT+ Complex and Neighbouring Visual Areas | 21 | 3 | 64 | 9 | 92 | 15 |
| V3B | Dorsal Stream Visual Cortex | 13 | 2 | 43 | 4 | 120 | 21 |
| STSdp | Auditory Association Cortex | 37 | 12 | 84 | 13 | 55 | 10 |
| IP0 | Inferior Parietal Cortex | 18 | 2 | 56 | 3 | 99 | 12 |
| 7PL | Superior Parietal Cortex | 28 | 6 | 58 | 11 | 73 | 11 |
| 31pd | Medial Parietal Cortex (including Posterior Cingulate) | 25 | 5 | 55 | 6 | 76 | 14 |
| MIP | Superior Parietal Cortex | 17 | 4 | 52 | 8 | 80 | 10 |
| 7Am | Superior Parietal Cortex | 23 | 4 | 53 | 9 | 63 | 12 |
| LO1 | MT+ Complex and Neighbouring Visual Areas | 12 | 3 | 38 | 8 | 83 | 18 |
| PCV | Medial Parietal Cortex (including Posterior Cingulate) | 23 | 5 | 47 | 9 | 60 | 13 |
| TPOJ3 | Temporo-Parieto-Occipital Junction | 15 | 2 | 51 | 4 | 64 | 10 |
| IP1 | Inferior Parietal Cortex | 15 | 3 | 43 | 3 | 60 | 7 |
| TPOJ2 | Temporo-Parieto-Occipital Junction | 16 | 3 | 48 | 6 | 40 | 5 |
| 52 | Insular and Frontal Opercular Cortex | 18 | 2 | 41 | 3 | 40 | 8 |
| TPOJ1 | Temporo-Parieto-Occipital Junction | 17 | 3 | 46 | 5 | 34 | 6 |
| STGa | Auditory Association Cortex | 31 | 7 | 45 | 5 | 19 | 3 |
| A5 | Auditory Association Cortex | 19 | 3 | 39 | 4 | 32 | 7 |
| 31pv | Medial Parietal Cortex (including Posterior Cingulate) | 14 | 2 | 32 | 3 | 41 | 5 |
| VIP | Superior Parietal Cortex | 12 | 3 | 31 | 5 | 38 | 5 |
| 7AL | Superior Parietal Cortex | 10 | 2 | 30 | 5 | 38 | 8 |
| A4 | Auditory Association Cortex | 14 | 3 | 34 | 4 | 30 | 6 |
| 33pr | Anterior Cingulate and Medial Prefrontal Cortex | 14 | 2 | 28 | 4 | 33 | 5 |
| LIPv | Superior Parietal Cortex | 8 | 2 | 28 | 5 | 37 | 8 |
| 4 | Somatosensory and Motor Cortex | 8 | 2 | 28 | 5 | 26 | 5 |
| PF | Inferior Parietal Cortex | 11 | 3 | 27 | 5 | 21 | 3 |
| 2 | Somatosensory and Motor Cortex | 7 | 1 | 25 | 4 | 23 | 3 |
| PSL | Temporo-Parieto-Occipital Junction | 9 | 1 | 24 | 3 | 18 | 2 |
| a24pr | Anterior Cingulate and Medial Prefrontal Cortex | 10 | 2 | 18 | 2 | 21 | 3 |
| p32pr | Anterior Cingulate and Medial Prefrontal Cortex | 12 | 3 | 18 | 3 | 20 | 4 |
| PBelt | Early Auditory Cortex | 9 | 2 | 21 | 2 | 19 | 4 |
| 7PC | Superior Parietal Cortex | 6 | 2 | 20 | 4 | 22 | 4 |
| STV | Temporo-Parieto-Occipital Junction | 7 | 2 | 23 | 3 | 18 | 3 |
| 23d | Medial Parietal Cortex (including Posterior Cingulate) | 7 | 1 | 18 | 3 | 22 | 3 |
| a32pr | Anterior Cingulate and Medial Prefrontal Cortex | 11 | 3 | 18 | 3 | 17 | 3 |
| LBelt | Early Auditory Cortex | 7 | 1 | 19 | 2 | 19 | 3 |
| MBelt | Early Auditory Cortex | 6 | 1 | 19 | 2 | 17 | 4 |
| 23c | Medial Parietal Cortex (including Posterior Cingulate) | 6 | 1 | 15 | 2 | 21 | 3 |
| 8BM | Anterior Cingulate and Medial Prefrontal Cortex | 10 | 2 | 16 | 3 | 15 | 3 |
| p24pr | Anterior Cingulate and Medial Prefrontal Cortex | 7 | 1 | 15 | 2 | 19 | 3 |
| 31a | Medial Parietal Cortex (including Posterior Cingulate) | 7 | 2 | 14 | 1 | 20 | 3 |
| 5mv | Paracentral Lobular and Mid Cingulate Cortex | 5 | 1 | 15 | 2 | 20 | 4 |
| 1 | Somatosensory and Motor Cortex | 4 | 1 | 19 | 3 | 16 | 3 |
| LIPd | Superior Parietal Cortex | 4 | 1 | 14 | 3 | 20 | 4 |
| AAIC | Insular and Frontal Opercular Cortex | 12 | 4 | 17 | 3 | 7 | 1 |
| PoI2 | Insular and Frontal Opercular Cortex | 13 | 5 | 14 | 3 | 8 | 1 |
| p24 | Anterior Cingulate and Medial Prefrontal Cortex | 7 | 2 | 13 | 2 | 15 | 2 |
| RI | Early Auditory Cortex | 4 | 1 | 18 | 1 | 13 | 2 |
| 5L | Paracentral Lobular and Mid Cingulate Cortex | 5 | 1 | 12 | 3 | 17 | 4 |
| 3b | Somatosensory and Motor Cortex | 3 | 1 | 15 | 3 | 15 | 3 |
| TA2 | Auditory Association Cortex | 8 | 2 | 15 | 3 | 11 | 2 |
| IP2 | Inferior Parietal Cortex | 4 | 1 | 14 | 3 | 15 | 3 |
| SCEF | Paracentral Lobular and Mid Cingulate Cortex | 5 | 1 | 13 | 2 | 14 | 3 |
| 9m | Anterior Cingulate and Medial Prefrontal Cortex | 8 | 2 | 12 | 3 | 11 | 2 |
| A1 | Early Auditory Cortex | 4 | 1 | 12 | 1 | 13 | 3 |
| d32 | Anterior Cingulate and Medial Prefrontal Cortex | 7 | 1 | 10 | 2 | 11 | 2 |
| 6mp | Paracentral Lobular and Mid Cingulate Cortex | 3 | 1 | 12 | 3 | 12 | 2 |
| 6ma | Paracentral Lobular and Mid Cingulate Cortex | 3 | 1 | 10 | 2 | 13 | 2 |
| SFL | DorsoLateral Prefrontal Cortex | 4 | 1 | 7 | 1 | 10 | 2 |
| 3a | Somatosensory and Motor Cortex | 2 | 1 | 11 | 2 | 8 | 1 |
| AIP | Superior Parietal Cortex | 3 | 1 | 8 | 1 | 10 | 2 |
| 24dv | Paracentral Lobular and Mid Cingulate Cortex | 4 | 2 | 6 | 2 | 10 | 4 |
| 6r | Premotor Cortex | 3 | 1 | 9 | 1 | 6 | 1 |
| 8BL | DorsoLateral Prefrontal Cortex | 4 | 1 | 7 | 2 | 8 | 1 |
| PFcm | Posterior Opercular Cortex | 3 | 1 | 8 | 1 | 7 | 1 |
| 8Av | DorsoLateral Prefrontal Cortex | 3 | 1 | 7 | 1 | 7 | 1 |
| 24dd | Paracentral Lobular and Mid Cingulate Cortex | 2 | 0 | 6 | 1 | 8 | 2 |
| 6a | Premotor Cortex | 2 | 0 | 7 | 1 | 7 | 1 |
| 6d | Premotor Cortex | 2 | 1 | 8 | 2 | 6 | 1 |
| a24 | Anterior Cingulate and Medial Prefrontal Cortex | 3 | 1 | 5 | 1 | 6 | 2 |
| 6v | Premotor Cortex | 2 | 1 | 6 | 1 | 5 | 1 |
| PFt | Inferior Parietal Cortex | 2 | 1 | 7 | 1 | 4 | 1 |
| 44 | Inferior Frontal Cortex | 2 | 1 | 5 | 1 | 4 | 1 |
| FEF | Premotor Cortex | 2 | 1 | 5 | 1 | 5 | 1 |
| 5m | Somatosensory and Motor Cortex | 2 | 0 | 4 | 1 | 5 | 1 |
| 55b | Premotor Cortex | 2 | 0 | 5 | 1 | 4 | 1 |
| PFop | Inferior Parietal Cortex | 2 | 0 | 5 | 1 | 4 | 1 |
| 8C | DorsoLateral Prefrontal Cortex | 2 | 0 | 5 | 1 | 3 | 1 |
| i6-8 | DorsoLateral Prefrontal Cortex | 2 | 0 | 4 | 1 | 4 | 1 |
| 9p | DorsoLateral Prefrontal Cortex | 1 | 0 | 4 | 1 | 4 | 1 |
| 10d | Orbital and Polar Frontal Cortex | 2 | 1 | 3 | 1 | 3 | 1 |
| 9a | DorsoLateral Prefrontal Cortex | 1 | 0 | 3 | 1 | 4 | 1 |
| OP1 | Posterior Opercular Cortex | 1 | 0 | 4 | 1 | 2 | 1 |
| IFJa | Inferior Frontal Cortex | 2 | 0 | 3 | 1 | 3 | 1 |
| 9-46d | DorsoLateral Prefrontal Cortex | 1 | 0 | 2 | 0 | 4 | 0 |
| 8Ad | DorsoLateral Prefrontal Cortex | 1 | 0 | 2 | 0 | 4 | 1 |
| 46 | DorsoLateral Prefrontal Cortex | 1 | 0 | 3 | 1 | 3 | 1 |
| IFSp | Inferior Frontal Cortex | 1 | 0 | 3 | 1 | 2 | 0 |
| s6-8 | DorsoLateral Prefrontal Cortex | 1 | 0 | 2 | 0 | 4 | 1 |
| p9-46v | DorsoLateral Prefrontal Cortex | 1 | 0 | 2 | 1 | 3 | 1 |
| OP4 | Posterior Opercular Cortex | 1 | 1 | 3 | 1 | 2 | 0 |
| a47r | Orbital and Polar Frontal Cortex | 1 | 0 | 2 | 1 | 3 | 1 |
| 43 | Posterior Opercular Cortex | 1 | 0 | 3 | 0 | 2 | 0 |
| Ig | Insular and Frontal Opercular Cortex | 1 | 0 | 3 | 0 | 2 | 0 |
| MI | Insular and Frontal Opercular Cortex | 1 | 0 | 3 | 1 | 2 | 1 |
| 47s | Orbital and Polar Frontal Cortex | 1 | 0 | 3 | 1 | 2 | 1 |
| PEF | Premotor Cortex | 1 | 0 | 2 | 0 | 2 | 1 |
| p32 | Anterior Cingulate and Medial Prefrontal Cortex | 1 | 0 | 2 | 0 | 2 | 1 |
| IFSa | Inferior Frontal Cortex | 1 | 0 | 2 | 0 | 2 | 0 |
| 45 | Inferior Frontal Cortex | 1 | 0 | 2 | 0 | 2 | 0 |
| 13l | Orbital and Polar Frontal Cortex | 1 | 0 | 2 | 1 | 1 | 0 |
| pOFC | Orbital and Polar Frontal Cortex | 1 | 0 | 2 | 1 | 1 | 0 |
| FOP4 | Insular and Frontal Opercular Cortex | 1 | 0 | 2 | 0 | 2 | 0 |
| p10p | Orbital and Polar Frontal Cortex | 1 | 0 | 1 | 0 | 2 | 0 |
| OFC | Orbital and Polar Frontal Cortex | 1 | 0 | 2 | 1 | 1 | 0 |
| a9-46v | DorsoLateral Prefrontal Cortex | 1 | 0 | 2 | 0 | 1 | 0 |
| OP2-3 | Posterior Opercular Cortex | 1 | 0 | 2 | 0 | 1 | 0 |
| FOP1 | Posterior Opercular Cortex | 0 | 0 | 2 | 1 | 1 | 0 |
| IFJp | Inferior Frontal Cortex | 1 | 0 | 1 | 0 | 1 | 0 |
| p47r | Inferior Frontal Cortex | 1 | 0 | 1 | 0 | 1 | 0 |
| 10r | Anterior Cingulate and Medial Prefrontal Cortex | 1 | 0 | 1 | 0 | 1 | 0 |
| 10pp | Orbital and Polar Frontal Cortex | 1 | 0 | 1 | 0 | 1 | 0 |
| AVI | Insular and Frontal Opercular Cortex | 1 | 0 | 1 | 1 | 1 | 0 |
| 11l | Orbital and Polar Frontal Cortex | 1 | 0 | 1 | 0 | 1 | 0 |
| 10v | Anterior Cingulate and Medial Prefrontal Cortex | 1 | 0 | 1 | 0 | 1 | 0 |
| 47l | Inferior Frontal Cortex | 0 | 0 | 1 | 0 | 1 | 0 |
| a10p | Orbital and Polar Frontal Cortex | 0 | 0 | 1 | 0 | 1 | 0 |
| FOP5 | Insular and Frontal Opercular Cortex | 0 | 0 | 1 | 0 | 1 | 1 |
| 25 | Anterior Cingulate and Medial Prefrontal Cortex | 0 | 0 | 1 | 0 | 1 | 0 |
| FOP3 | Insular and Frontal Opercular Cortex | 0 | 0 | 1 | 0 | 1 | 0 |
| FOP2 | Insular and Frontal Opercular Cortex | 0 | 0 | 1 | 0 | 1 | 0 |
| 47m | Orbital and Polar Frontal Cortex | 0 | 0 | 1 | 0 | 1 | 0 |
| s32 | Anterior Cingulate and Medial Prefrontal Cortex | 0 | 0 | 1 | 0 | 0 | 0 |
