# Supplementary material for: New insights into anatomical connectivity along the anterior–posterior axis of the human hippocampus using in vivo quantitative fibre tracking
Source: eLife. 2022 Nov 8;11:e76143. doi: 10.7554/eLife.76143 (PMC9643002; doi:10.7554/eLife.76143)
Supplement: Supplementary file 3. [file elife-76143-supp3.docx]

**Supplementary File 3. List of abbreviations for all cortical brain areas in the Human Connectome Project Multi-Modal Parcellation (HCPMMP) scheme.**

| **Brain region** | **Cortical areas within each brain region** | |
| --- | --- | --- |
| Medial Temporal Cortex | EC | Entorhinal Cortex |
|  | PeEc | Perirhinal Ectorhinal Cortex |
|  | PHA1 | ParaHippocampal Area 1 |
|  | PHA2 | ParaHippocampal Area 2 |
|  | PHA3 | ParaHippocampal Area 3 |
| Lateral Temporal Cortex (including Temporal Pole) | PHT | Area PHT |
|  | TE1a | Area TE1 anterior |
|  | TE1m | Area TE1 Middle |
|  | TE1p | Area TE1 posterior |
|  | TE2a | Area TE2 anterior |
|  | TE2p | Area TE2 posterior |
|  | TF | Area TF |
|  | TGd | Area TG dorsal |
|  | TGv | Area TG Ventral |
| Medial Parietal Cortex (including Posterior Cingulate) | 23c | Area 23c |
|  | 23d | Area 23d |
|  | 31a | Area 31a |
|  | 31pd | Area 31pd |
|  | 31pv | Area 31p ventral |
|  | 7m | Area 7m |
|  | d23ab | Area dorsal 23 a+b |
|  | DVT | Dorsal Transitional Visual Area |
|  | PCV | PreCuneus Visual Area |
|  | POS1 | Parieto-Occipital Sulcus Area 1 |
|  | POS2 | Parieto-Occipital Sulcus Area 2 |
|  | ProS | ProStriate Area |
|  | RSC | RetroSplenial Complex |
|  | v23ab | Area ventral 23 a+b |
| Early Visual Cortex (Occipital) | V1 | Primary Visual Cortex |
|  | V2 | Second Visual Area |
|  | V3 | Third Visual Area |
|  | V4 | Fourth Visual Area |
| Ventral Stream Visual Cortex | FFC | Fusiform Face Complex |
|  | PIT | Posterior InferoTemporal Complex |
|  | V8 | Eighth Visual Area |
|  | VMV1 | VentroMedial Visual Area 1 |
|  | VMV2 | VentroMedial Visual Area 2 |
|  | VMV3 | VentroMedial Visual Area 3 |
|  | VVC | Ventral Visual Complex |
| Dorsal Stream Visual Cortex | IPS1 | IntraParietal Sulcus Area 1 |
|  | V3A | Area V3A |
|  | V3B | Area V3B |
|  | V6 | Sixth Visual Area |
|  | V6A | Area V6A |
|  | V7 | Seventh Visual Area |
| MT+ Complex and Neighbouring Visual Areas | FST | Area FST |
|  | LO1 | Area Lateral Occipital 1 |
|  | LO2 | Area Lateral Occipital 2 |
|  | LO3 | Area Lateral Occipital 3 |
|  | MST | Medial Superior Temporal Area |
|  | MT | Middle Temporal Area |
|  | PH | Area PH |
|  | V3CD | Area V3CD |
|  | V4t | Area V4t |
| Inferior Parietal Cortex | IP0 | Area IntraParietal 0 |
|  | IP1 | Area IntraParietal 1 |
|  | IP2 | Area IntraParietal 2 |
|  | PF | Area PF Complex |
|  | PFm | Area PFm Complex |
|  | PFop | Area PF opercular |
|  | PFt | Area PFt |
|  | PGi | Area PGi |
|  | PGp | Area PGp |
|  | PGs | Area PGs |
| Auditory Association Cortex | A4 | Auditory 4 Complex |
|  | A5 | Auditory 5 Complex |
|  | STGa | Area STGa |
|  | STSda | Area STSd anterior |
|  | STSdp | Area STSd posterior |
|  | STSva | Area STSv anterior |
|  | STSvp | Area STSv posterior |
|  | TA2 | Area TA2 |
| Insular and Frontal Opercular Cortex | 52 | Area 52 |
|  | AAIC | Anterior Agranular Insula Complex |
|  | AVI | Anterior Ventral Insular Area |
|  | FOP2 | Frontal Opercular Area 2 |
|  | FOP3 | Frontal Opercular Area 3 |
|  | FOP4 | Frontal Opercular Area 4 |
|  | FOP5 | Area Frontal Opercular 5 |
|  | Ig | Insular Granular Complex |
|  | MI | Middle Insular Area |
|  | PI | Para-Insular Area |
|  | Pir | Pirform Cortex |
|  | PoI1 | Area Posterior Insular 1 |
|  | PoI2 | Posterior Insular Area 2 |
| Superior Parietal Cortex | 7AL | Lateral Area 7A |
|  | 7Am | Medial Area 7A |
|  | 7PC | Area 7PC |
|  | 7PL | Lateral Area 7P |
|  | 7Pm | Medial Area 7P |
|  | AIP | Anterior IntraParietal Area |
|  | LIPd | Area Lateral IntraParietal dorsal |
|  | LIPv | Area Lateral IntraParietal ventral |
|  | MIP | Medial IntraParietal Area |
|  | VIP | Ventral IntraParietal Complex |
| Temporo-Parieto-Occipital Junction | PSL | PeriSylvian Language Area |
|  | STV | Superior Temporal Visual Area |
|  | TPOJ1 | Area TemporoParietoOccipital Junction 1 |
|  | TPOJ2 | Area TemporoParietoOccipital Junction 2 |
|  | TPOJ3 | Area TemporoParietoOccipital Junction 3 |
| Anterior Cingulate and Medial Prefrontal Cortex | 25 | Area 25 |
|  | 10r | Area 10r |
|  | 10v | Area 10v |
|  | 33pr | Area 33 prime |
|  | 8BM | Area 8BM |
|  | 9m | Area 9 Middle |
|  | a24 | Area a24 |
|  | a24pr | Anterior 24 prime |
|  | a32pr | Area anterior 32 prime |
|  | d32 | Area dorsal 32 |
|  | p24 | Area posterior 24 |
|  | p24pr | Area Posterior 24 prime |
|  | p32 | Area p32 |
|  | p32pr | Area p32 prime |
|  | s32 | Area s32 |
| Somatosensory and Motor Cortex | 1 | Area 1 |
|  | 2 | Area 2 |
|  | 4 | Primary Motor Cortex |
|  | 3a | Area 3a |
|  | 3b | Primary Sensory Cortex |
|  | 5m | Area 5m |
| Early Auditory Cortex | A1 | Primary Auditory Cortex |
|  | LBelt | Lateral Belt Complex |
|  | MBelt | Medial Belt Complex |
|  | PBelt | ParaBelt Complex |
|  | RI | RetroInsular Cortex |
| Paracentral Lobular and Mid Cingulate Cortex | 24dd | Dorsal Area 24d |
|  | 24dv | Ventral Area 24d |
|  | 5mv | Area 5m ventral |
|  | 5L | Area 5L |
|  | 6ma | Area 6m anterior |
|  | 6mp | Area 6mp |
|  | SCEF | Supplementary and Cingulate Eye Field |
| Dorsolateral Prefrontal Cortex | 46 | Area 46 |
|  | 8Ad | Area 8Ad |
|  | 8Av | Area 8Av |
|  | 8BL | Area 8B Lateral |
|  | 8C | Area 8C |
|  | 9-46d | Area 9-46d |
|  | 9a | Area 9 anterior |
|  | 9p | Area 9 Posterior |
|  | a9-46v | Area anterior 9-46v |
|  | i6-8 | Inferior 6-8 Transitional Area |
|  | p9-46v | Area posterior 9-46v |
|  | s6-8 | Superior 6-8 Transitional Area |
|  | SFL | Superior Frontal Language Area |
| Premotor Cortex | 55b | Area 55b |
|  | 6a | Area 6 anterior |
|  | 6d | Dorsal area 6 |
|  | 6r | Rostral Area 6 |
|  | 6v | Ventral Area 6 |
|  | FEF | Frontal Eye Fields |
|  | PEF | Premotor Eye Field |
| Orbital and Polar Frontal Cortex | 10d | Area 10d |
|  | 10pp | Polar 10p |
|  | 11l | Area 11l |
|  | 13l | Area 13l |
|  | 47m | Area 47m |
|  | 47s | Area 47s |
|  | a10p | Area anterior 10p |
|  | a47r | Area anterior 47r |
|  | OFC | Orbital Frontal Complex |
|  | p10p | Area posterior 10p |
|  | pOFC | posterior OFC Complex |
| Inferior Frontal Cortex | 44 | Area 44 |
|  | 45 | Area 45 |
|  | 47l | Area 47l (47 lateral) |
|  | IFJa | Area IFJa |
|  | IFJp | Area IFJp |
|  | IFSa | Area IFSa |
|  | IFSp | Area IFSp |
|  | p47r | Area posterior 47r |
| Posterior Opercular Cortex | 43 | Area 43 |
|  | FOP1 | Frontal Opercular Area 1 |
|  | OP1 | Area OP1/SII |
|  | OP2-3 | Area OP2-3/VS |
|  | OP4 | Area OP4/PV |
|  | PFcm | Area PFcm |
