# Supplementary material for: New insights into anatomical connectivity along the anterior–posterior axis of the human hippocampus using in vivo quantitative fibre tracking
Source: eLife. 2022 Nov 8;11:e76143. doi: 10.7554/eLife.76143 (PMC9643002; doi:10.7554/eLife.76143)
Supplement: Supplementary file 4. — Column 1 displays cortical areas as defined by the Human Connectome Project Multi-Modal Parcellation (HCPMMP) scheme and ordered by strength of connectivity with the whole hippocampus (abbreviations for all cortical areas are defined in Supplementary file 3). Column 2 designates the portion of hippocampus (head, body, tail). Column 3 displays the mean SIFT2-weighted value (connectivity strength) between each cortical area and the head, body, and tail of the hippocampus. Column 4 displays the standard error of the mean. Column 5 displays the contrast for each paired-samples t-test. Column 6 displays the t-statistic associated with each pair. Column 7 displays the p-value associated with each pair. Column 8 indicates the significance level associated with each pair following Bonferroni correction. ***<0.001, **<0.01, *<0.05, ns, not statistically significant. [file elife-76143-supp4.docx]

**Supplementary File 4. Connectivity between the twenty most highly connected cortical areas and the head, body and tail of the hippocampus.** Column 1 displays cortical areas as defined by the Human Connectome Project Multi-Modal Parcellation (HCPMMP) scheme and ordered by strength of connectivity with the whole hippocampus (abbreviations for all cortical areas are defined in Supplementary Table S3). Column 2 designates the portion of hippocampus (head, body, tail). Column 3 displays the mean SIFT2 weighted value (connectivity strength) between each cortical area and the head, body and tail of the hippocampus. Column 4 displays the standard error of the mean. Column 5 displays the contrast for each paired samples t-test. Column 6 displays the t-statistic associated with each pair. Column 7 displays the p-value associated with each pair. Column 8 indicates the significance level associated with each pair following Bonferroni correction. *** = <0.001, ** = <0.01, * = <0.05, n.s. = not statistically significant.

| **Cortical area** | **Portion of hippocampus** | **Mean SIFT2 weighted value (connectivity strength; n=10)** | **SE of Mean** | **Paired samples t-test** | | | |
| --- | --- | --- | --- | --- | --- | --- | --- |
|  |  |  |  | **Pair** | ***t*-statistic**  ***t*(9)** | ***p*-value** | **Significance** |
| TF | Head | 3287 | 500 | Head - body | -0.34 | 0.741 | n.s. |
|  | Body | 3452 | 410 | Body - tail | 9.077 | <0.001 | *** |
|  | Tail | 934 | 144 | Head - tail | 5.049 | 0.001 | ** |
| ProS | Head | 716 | 116 | Head - body | -5.75 | <0.001 | *** |
|  | Body | 1625 | 194 | Body - tail | -3.618 | 0.006 | ** |
|  | Tail | 3143 | 553 | Head - tail | -4.799 | 0.001 | ** |
| V1 | Head | 567 | 70 | Head - body | -9.508 | <0.001 | *** |
|  | Body | 1389 | 112 | Body - tail | -4.629 | 0.001 | ** |
|  | Tail | 3429 | 489 | Head - tail | -5.919 | <0.001 | *** |
| V2 | Head | 428 | 62 | Head - body | -11.910 | <0.001 | *** |
|  | Body | 1054 | 82 | Body - tail | -3.903 | 0.004 | ** |
|  | Tail | 2358 | 376 | Head - tail | -5.396 | <0.001 | *** |
| POS1 | Head | 620 | 89 | Head - body | -6.255 | <0.001 | *** |
|  | Body | 1299 | 112 | Body - tail | -2.196 | 0.056 | n.s. |
|  | Tail | 1793 | 297 | Head - tail | -4.043 | 0.003 | ** |
| TGd | Head | 1576 | 182 | Head - body | 0.79 | 0.450 | n.s. |
|  | Body | 1446 | 117 | Body - tail | 11.378 | <0.001 | *** |
|  | Tail | 443 | 47 | Head - tail | 6.641 | <0.001 | *** |
| TGv | Head | 1589 | 207 | Head - body | 1.121 | 0.291 | n.s. |
|  | Body | 1358 | 144 | Body - tail | 8.211 | <0.001 | *** |
|  | Tail | 389 | 41 | Head - tail | 5.973 | <0.001 | *** |
| V3 | Head | 327 | 37 | Head - body | -7.204 | <0.001 | *** |
|  | Body | 810 | 83 | Body - tail | -3.921 | 0.004 | ** |
|  | Tail | 1942 | 357 | Head - tail | -4.718 | 0.001 | ** |
| TE2a | Head | 754 | 132 | Head - body | -3.759 | 0.004 | ** |
|  | Body | 1038 | 107 | Body - tail | 11.479 | <0.001 | *** |
|  | Tail | 422 | 65 | Head - tail | 3.787 | 0.004 | ** |
| VMV2 | Head | 354 | 68 | Head - body | -7.919 | <0.001 | *** |
|  | Body | 860 | 111 | Body - tail | -0.375 | 0.716 | n.s. |
|  | Tail | 891 | 97 | Head - tail | -6.248 | <0.001 | *** |
| RSC | Head | 401 | 55 | Head - body | -8.647 | <0.001 | *** |
|  | Body | 733 | 52 | Body - tail | -1.55 | 0.155 | n.s. |
|  | Tail | 928 | 107 | Head - tail | -3.727 | 0.005 | ** |
| VVC | Head | 460 | 76 | Head - body | -5.554 | <0.001 | *** |
|  | Body | 969 | 129 | Body - tail | 4.058 | 0.003 | ** |
|  | Tail | 528 | 48 | Head - tail | -0.885 | 0.399 | n.s. |
| DVT | Head | 247 | 36 | Head - body | -5.436 | <0.001 | *** |
|  | Body | 515 | 65 | Body - tail | -3.374 | 0.008 | ** |
|  | Tail | 1178 | 253 | Head - tail | -3.954 | 0.003 | ** |
| POS2 | Head | 303 | 32 | Head - body | -6.682 | <0.001 | *** |
|  | Body | 590 | 47 | Body - tail | -2.02 | 0.074 | n.s. |
|  | Tail | 909 | 195 | Head - tail | -3.211 | 0.011 | * |
| VMV1 | Head | 245 | 47 | Head - body | -5.392 | <0.001 | *** |
|  | Body | 644 | 115 | Body - tail | -2.939 | 0.017 | n.s. (following Bonferroni correction) |
|  | Tail | 899 | 110 | Head - tail | -7.372 | <0.001 | *** |
| FFC | Head | 355 | 49 | Head - body | -5.364 | <0.001 | *** |
|  | Body | 798 | 109 | Body - tail | 2.805 | 0.021 | n.s. (following Bonferroni correction) |
|  | Tail | 517 | 55 | Head - tail | -2.24 | 0.052 | n.s. |
| V4 | Head | 183 | 20 | Head - body | -10.472 | <0.001 | *** |
|  | Body | 472 | 40 | Body - tail | -4.002 | 0.003 | ** |
|  | Tail | 946 | 116 | Head - tail | -6.136 | <0.001 | *** |
| TE2p | Head | 348 | 69 | Head - body | -5.453 | <0.001 | *** |
|  | Body | 658 | 101 | Body - tail | 5.168 | 0.001 | ** |
|  | Tail | 282 | 45 | Head - tail | 1.163 | 0.275 | n.s. |
| V6 | Head | 113 | 16 | Head - body | -5.428 | <0.001 | *** |
|  | Body | 259 | 37 | Body - tail | -3.652 | 0.005 | ** |
|  | Tail | 678 | 149 | Head - tail | -4.047 | 0.003 | ** |
| V3A | Head | 103 | 11 | Head - body | -5.641 | <0.001 | *** |
|  | Body | 252 | 35 | Body - tail | -3.46 | 0.007 | ** |
|  | Tail | 674 | 155 | Head - tail | -3.895 | 0.004 | ** |
