# Supplementary material for: New insights into anatomical connectivity along the anterior–posterior axis of the human hippocampus using in vivo quantitative fibre tracking
Source: eLife. 2022 Nov 8;11:e76143. doi: 10.7554/eLife.76143 (PMC9643002; doi:10.7554/eLife.76143)
Supplement: Transparent reporting form [file elife-76143-transrepform1.pdf]

1st Floor
24 Hills Road
Cambridge CB2 1JP, UK

P 01223 855340
W elifesciences.org
T @elife

# *eLife's* transparent reporting form

We encourage authors to provide detailed information *within their submission* to facilitate the interpretation and replication of experiments. Authors can upload supporting documentation to indicate the use of appropriate reporting guidelines for health-related research (see EQUATOR Network), life science research (see the BioSharing Information Resource), or the ARRIVE guidelines for reporting work involving animal research. Where applicable, authors should refer to any relevant reporting standards documents in this form.

If you have any questions, please consult our Journal Policies and/or contact us: editorial@elifesciences.org.

**Sample-size estimation**

- You should state whether an appropriate sample size was computed when the study was being designed
- You should state the statistical method of sample size computation and any required assumptions
- If no explicit power analysis was used, you should describe how you decided what sample (replicate) size (number) to use

Please outline where this information can be found within the submission (e.g., sections or figure legends), or explain why this information doesn't apply to your submission:

No power analyses were carried out to determine the number of subjects to include in the study. The number of subjects (n=10) was selected as a balance between increasing statistical power for group metrics and the substantial manual involvement required for the analysis of each individual subject. As described in the manuscript, to ensure careful characterisation of hippocampal connectivity, a number of time-consuming manual steps were required, including manual segmentation of the hippocampus (see Materials and Methods; p23-24: lines 628-649) and manual editing of the grey matter-white matter interface adjacent to the hippocampus (see Materials and Methods; p24-25: 667-691). Furthermore, in our experience based on previous studies involving hippocampal measures that require considerable manual intervention, including a large number of cases can come at the expense of decreasing data reliability, for example, due to operator fatigue.

In addition, the 10 participants included in the study were selected to ensure the best quality T1 weighted data were used, where the hippocampus was clearly visible along its entire anterior-posterior extent. This is also described in the Materials and Methods section (Page 23: lines 613-616).

**Replicates**

- You should report how often each experiment was performed
- You should include a definition of biological versus technical replication
- The data obtained should be provided and sufficient information should be provided to indicate the number of independent biological and/or technical replicates
- If you encountered any outliers, you should describe how these were handled
- Criteria for exclusion/inclusion of data should be clearly stated
- High-throughput sequence data should be uploaded before submission, with a private link for reviewers provided (these are available from both GEO and ArrayExpress)

eLife Sciences Publications, Ltd is a limited liability non-profit non-stock corporation incorporated in the State of Delaware, USA, with company number 5030732, and is registered in the UK with company number FC030576 and branch number BR015634 at the address 1st Floor, 24 Hills Road, Cambridge CB2 1JP | August 2014

Please outline where this information can be found within the submission (e.g., sections or figure legends), or explain why this information doesn't apply to your submission:

No replicates were considered in our study besides the replicates involved within the generation of the multi-million number of probabilistic streamlines that are computed to construct the tractograms for each diffusion MRI dataset.

Also, no data exclusion took place after image analysis. The only exclusion took place during the initial selection of the 10 subjects (before fibre-tracking had been carried out). This was based on excluding subjects that had lower T1 weighted image quality, as described in our response to "Sample-size estimation" above.

**Statistical reporting**

- Statistical analysis methods should be described and justified
- Raw data should be presented in figures whenever informative to do so (typically when N per group is less than 10)
- For each experiment, you should identify the statistical tests used, exact values of N, definitions of center, methods of multiple test correction, and dispersion and precision measures (e.g., mean, median, SD, SEM, confidence intervals; and, for the major substantive results, a measure of effect size (e.g., Pearson's r, Cohen's d)
- Report exact p-values wherever possible alongside the summary statistics and 95% confidence intervals. These should be reported for all key questions and not only when the p-value is less than 0.05.

Please outline where this information can be found within the submission (e.g., sections or figure legends), or explain why this information doesn't apply to your submission:

To assess whether connectivity values between each cortical brain area and the head, body and tail portions of the hippocampus were statistically significant, we conducted Bonferroni-corrected paired-samples t-tests. These are reported in the Results section when significant at a level of $p < 0.05$ (see pages 8-9 of the main text: lines 177-207) and in Table S4 of the Supplementary Information which includes exact p-values (page 64-66). The mean and SEM relating to these analyses are also provided in table S4 of the Supplementary Information (page 64-66) and are presented graphically in Figure 1 (Page 6). We describe these Bonferroni-corrected paired-samples t-tests in the Materials and Methods section (page 26; lines 726-729).

(For large datasets, or papers with a very large number of statistical tests, you may upload a single table file with tests, Ns, etc., with reference to sections in the manuscript.)

**Group allocation**

- Indicate how samples were allocated into experimental groups (in the case of clinical studies, please specify allocation to treatment method); if randomization was used, please also state if restricted randomization was applied
- Indicate if masking was used during group allocation, data collection and/or data analysis

Please outline where this information can be found within the submission (e.g., sections or figure legends), or explain why this information doesn't apply to your submission:

Our study comprised only one group. We therefore did not require any experimental group allocations.

**Additional data files ("source data")**

- We encourage you to upload relevant additional data files, such as numerical data that are represented as a graph in a figure, or as a summary table
- Where provided, these should be in the most useful format, and they can be uploaded as "Source data" files linked to a main figure or table
- Include model definition files including the full list of parameters used
- Include code used for data analysis (e.g., R, MatLab)
- Avoid stating that data files are "available upon request"

Please indicate the figures or tables for which source data files have been provided:

We provide comprehensive lists of additional numerical data relating to the mean SIFT2 weighted connectivity values (connectivity strength) between the hippocampus and each cortical brain area of the Human Connectome Project Multi-Modal Parcellation and the SEM associated with each of these values. These are presented for the top 20 most highly connected cortical brain areas in Table 1 (p7) and are more comprehensively described in Supplemental Information Tables S1 (pages 44-51), S2 (pages 52-58) and S4 (pages 64-66).

We will provide all code used for data analysis in the Supplementary Materials if accepted for publication. This is noted in the Materials and Methods section (pages 26-27: lines 761-762) as a note to Editors and Reviewers (highlighted yellow).
